# Prospective Randomized Controlled Clinical Trial of the Long-Term Effects of Omeprazole on Healthy Dogs

**DOI:** 10.3390/ani14081168

**Published:** 2024-04-12

**Authors:** Laura Gil-Vicente, Germán Martín, Carme Soler, Anna Vila, María Rocío Saiz, Paula F. Navarro

**Affiliations:** 1 Facultad de Veterinaria y Ciencias Experimentales, Universidad Católica de Valencia San Vicente Mártir, 46001 Valencia, Spain; laura.gil@ucv.es (L.G.-V.); german.martin@ucv.es (G.M.); mdc.soler@ucv.es (C.S.); anna.vila@ucv.es (A.V.); mdr.saiz@ucv.es (M.R.S.); 2 Hospital Veterinario UCV, Universidad Católica de Valencia San Vicente Mártir, 46018 Valencia, Spain

**Keywords:** canine, proton pump inhibitor, omeprazole, gastrin, cobalamin, dysbiosis

## Abstract

**Simple Summary:**

In the present study, the effects of omeprazole on healthy dogs were investigated, and long-term (60 days) use of this medication led to a significant increase in serum gastrin levels. However, no notable changes in cobalamin levels were detected. Gastrin is a hormone involved in regulating gastric acid production, and its elevation suggests a potential risk of gastric hyperplasia or cancer. Notably, adverse effects were observed in 18% of the dogs receiving omeprazole. These findings align with previous research indicating that omeprazole can disrupt the gastrointestinal microbiome, potentially leading to digestive disturbances. While the short-term use of omeprazole is generally well-tolerated in dogs, caution is warranted regarding its prolonged administration due to the risk of gastrointestinal complications. Further studies are needed to explore the long-term effects of omeprazole and its implications for canine health.

**Abstract:**

The use of omeprazole as a preventive treatment for gastrointestinal ulcers in veterinary medicine has been questioned during previous years. The aim of the present study is to assess the long-term effect of omeprazole on cobalamin and serum gastrin levels in healthy dogs. Eighteen healthy dogs were included: 10 in the control group and 8 in the omeprazole group. Three samples were collected: before starting the treatment (T_0_), 30 days after the start of treatment (T_1_), and at 60 days (T_2_). The mean cobalamin value (ng/L) in the control group was 481.4 (±293.70) at T_0_, 481.4 (±170.21) at T_1_, and 513.2 (±174.50) at T_2_. In the omeprazole group, the values were 424.62 (±161.57) at T_0_, 454.5 (±160.96) at T_1_, and 414.87 (±127.90) at T_2_. No statistically significant changes were detected in cobalamin levels between the three-time period in both study groups. These results agree with previous findings in felines but contrast with human medicine studies. The median gastrin values (pg/mL) in the control group were 62.45 [30.17–218.75] at T_0_, 76.06 [30.67–199.87] at T_1_, and 63.02 [35.81–176.06] at T_2_. The median gastrin value in the omeprazole group was 67.59 [55.96–101.60] at T_0_, 191.77 [75.31–1901.77] at T_1_, and 128.16 [43.62–1066.46] at T_2_. Statistically significant differences were detected (*p* = 0.008), indicating an increase in gastrin levels after initiating treatment with omeprazole. In conclusion, the increased levels of gastrin observed in this population underscore the importance of conducting a comprehensive clinical assessment to identify potential gastrointestinal disorders, particularly in consideration of the usage of omeprazole as a preventive treatment.

## 1. Introduction

Proton pump inhibitors (PPIs), including omeprazole, pantoprazole, esomeprazole, and lansoprazole, constitute a class of gastroprotective drugs [1]. Omeprazole is routinely prescribed in veterinary medicine to mitigate the gastrointestinal mucosal injury associated with the administration of steroids or nonsteroidal anti-inflammatory drugs [2,3,4]. However, current guidelines do not recommend its routine use for managing gastritis, pancreatitis, liver disease, or kidney disease in dogs and cats without additional risk factors for ulceration or gastrointestinal bleeding, and the preventive administration of gastrointestinal protectants in healthy dogs is discouraged [1]. Its mechanism involves the inhibition of the H^+^/K^+^ ATPase pump in gastric parietal cells, resulting in the blockade of gastric acid secretion [5]. It is metabolized rapidly and completely by the liver, by cytochrome P_450_ enzymes (CYP_450_), and eliminated through the urine [1]. Administration can be either parenteral or enteral, with rapid absorption in the small intestine and peak plasmatic concentrations observed three hours post-administration [6].

In human medicine, prolonged omeprazole use has been linked to severe side effects, such as cobalamin (Vitamin B_12_) deficiency and calcium and magnesium homeostasis disruption, leading to conditions such as osteoporosis and pathological fractures [7]. The identified substantial deficiencies in vitamins (B_12_ and C) and minerals (calcium, magnesium, and iron) are linked to an increased gastric pH, leading to hypochlorhydria or achlorhydria following the use of PPIs [8].

In dogs and cats, PPIs, including omeprazole, have demonstrated efficacy in increasing gastric pH, prescribed for managing dyspepsia, peptic ulceration, and gastroesophageal reflux disease [1,9]. Short-term administration of PPIs is generally well-tolerated in dogs and cats, with minor adverse effects, primarily self-limiting diarrhea and intestinal dysbiosis [4,7,10].

In feline patients, long-term administration for 60 days can lead to hypergastrinemia and an increase in gastric acid release after disruption of the drug [11]. Gastric acid production is mainly regulated by gastrin, and the inhibition of the negative feedback occurs when hypochloridria is detected and serum gastrin levels increase [12]. In adult humans, hypochloridria, due to the long-term PPIs used, can induce hypergastrinemia, potentially leading to gastric polyps or cancer [8]. Studies in dogs determined that serum gastric levels were significantly higher in patients treated with PPIs compared to a control group. However, the administration time of the antacids was not standardized [13]. Currently, there have been no long-term studies on the use of PPIs in dogs similar to those conducted in cats. Therefore, side effects observed in humans, such as hypergastrinemia, occur in this species, as it has only been demonstrated in short-term studies in dogs, highlighting the need for the present study.

Correlations between the administration of PPIs and increased risk of suffering urinary, enteric, or respiratory infections due to altered gastric microbiota and increased gastric pH have been reported in dogs and humans [8,14,15]. Also, long-term PPI therapy leads to a higher risk of enteric infections by *Salmonella* spp. and *Campylobacter* spp. [15]. Omeprazole induces alterations in the gastric microbiome, potentially impacting *Helicobacter pylori* infections positively in humans, but such changes may elevate the risk of other gastrointestinal infections [1,14]. Notably, administering omeprazole twice daily to healthy dogs for 15 days resulted in quantifiable changes in the gastric, duodenal, and fecal microbiome, accompanied by a significant increase in gastric pH and altered bacterial composition. *Lactobacillus* bacteria were notably elevated in the duodenum and fecal samples in male dogs during omeprazole therapy without qualitative changes in fecal composition [6,14]. Changes in pharyngeal flora, including *Staphylococcus* spp., *Bacillus* spp., and *Pasteurella* spp. overgrowth, also have been observed during the 12-day course with omeprazole [16]. The use of antacids in dogs under anesthesia to prevent aspiration pneumonia remains unclear, as it may potentially increase bacterial colonization in the patient, consequently predisposing them to bacterial pneumonia [17].

This study aims to evaluate the long-term effect of omeprazole on cobalamin and serum gastrin levels in healthy dogs and to describe the complications detected from its use.

## 2. Materials and Methods

### 2.1. Study Population

The study was approved by the research and ethics committee of the Universidad Católica de Valencia San Vicente Mártir (Valencia, Spain; CEEAUCV2007 (#2312-0115)), and included eighteen healthy dogs selected from veterinary hospital staff and veterinary students.

Age, breed, and sex were randomly selected. Healthy dogs had to meet the following criteria: anamnesis and complete physical examination without abnormalities, no other medications at the time of the study, and laboratory findings (hematology, biochemistry, and urinalysis) within normal reference limits. Dogs were excluded if they had a history of gastrointestinal pathology, including vomiting, anorexia, and diarrhea, or if more than 10% of the body weight was lost during the study. Additionally, dogs that developed systemic diseases or required therapeutics capable of interfering with any measured parameters, except for routine anthelmintic and heartworm preventative treatments, were excluded. Dogs under 7 kg or above 35 kg were excluded in order to maintain the therapeutic dosage of 1 mg/kg twice per day (BID) in all dogs.

### 2.2. Study Design

**Figure 1:**
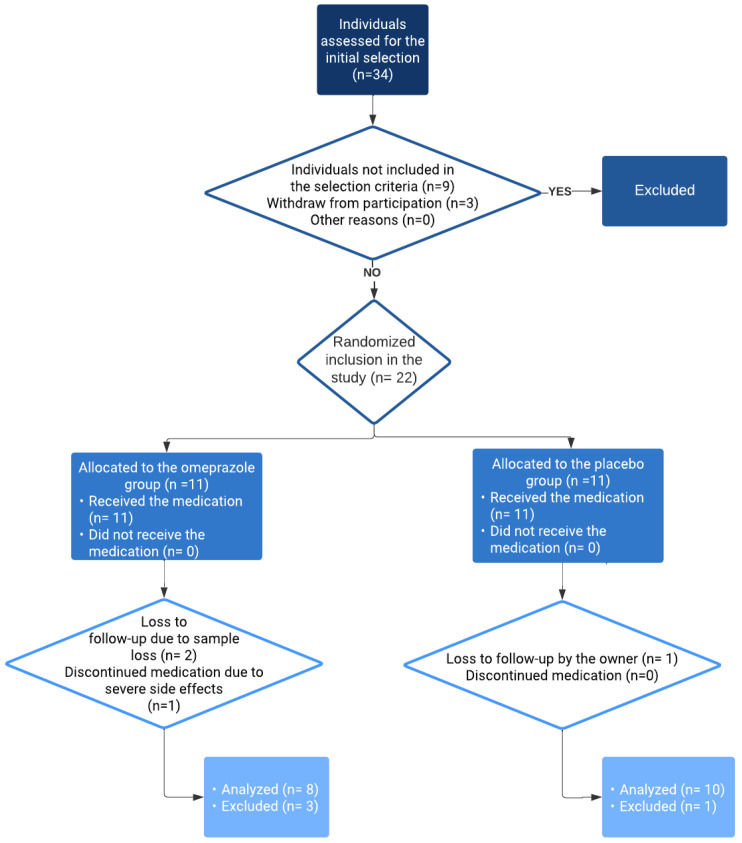
Flowchart depicting the progression through the phases of the randomized controlled clinical trial. The number of dogs that entered each phase of the study (*n*). Sixteen dogs were excluded throughout the study, and the time and reason for their exclusion are provided.

A randomized controlled clinical trial was conducted with two groups (control/study). Between July 2019 and July 2020, thirty-four dogs owned by employees and students of the university were prospectively examined to determine their inclusion in the study. Twenty-two dogs were examined for meeting the inclusion criteria, of which 18 dogs were analyzed. Inclusion in one of the two groups was randomized (Figure 1). The control group received a placebo (jelly capsules with 250 mg lactulose), and the study group received uncompounded omeprazole orally, with a median dose of 0.86 mg/kg (range: 0.53–1.0 mg/kg), administered through delayed-release capsules containing either 10 mg or 20 mg of omeprazole. Both groups followed a regimen of oral administration twice a day, 30 min before meals. For dogs in the study group, 10 mg omeprazole capsules were prescribed when the weight was between 7 kg and 15 kg, and 20 mg omeprazole capsules were prescribed when the weight was between 15 kg and 35 kg. All dogs were fed their regular food and had unlimited access to water.

### 2.3. Sample Collection

Blood samples were collected from the jugular or cephalic vein after a 12 h fast. Samples were collected in a 0.5 mL EDTA tube and two serum tubes, each with 2 mL capacity, and refrigerated at 4 °C until analysis. A reference laboratory (Idexx; Barcelona, Spain) processed all EDTA and serum samples. Complete blood count and biochemistry parameters such as glucose, blood urea nitrogen (BUN), creatinine, alanine aminotransferase (ALT), alkaline phosphatase (ALP), total solids, and albumin/globulin ratio were included. Two serum tubes were used to measure serum cobalamin and gastrin levels; these samples were centrifugated and refrigerated immediately in 1 mL microcentrifuge tubes at −20 °C until analysis. Analysis was performed by a reference laboratory (Idexx; Barcelona, Spain). Cobalamin and gastrin levels were analyzed using a chemiluminescence assay. Three samples were collected as follows: before starting the treatment (T_0_), 30 days after the start of treatment (T_1_), and at 60 days (T_2_).

Urine was collected by cystocentesis and stored at 4 °C until analysis. The urinalysis was performed in the hospital laboratory.

Owners were instructed to complete the questionnaire once per week. Data included the dog’s attitude, changes in appetite, or GI signs such as diarrhea, vomiting, or nausea. Fecal consistency was graded from 1 to 7 each day of each treatment using a standardized fecal scoring system (Fecal Scoring System, developed by Nestlé Purina PetCare Company, St. Louis, MN, USA). Diarrhea was defined as a fecal score > 4.

### 2.4. Statistical Analysis

The statistical analysis was performed using R version 3.4.3 software (R Development Core Team; Vienna, Austria). The sample size calculation was determined based on a prior study conducted in feline subjects, specifically focusing on the cobalamin outcome variable [7]. Normality was assessed using the Shapiro–Wilk test, considering non-normality if *p* < 0.05. The outliers were identified using box plots. These values were excluded if they were considered outside the study population. An ANOVA one-way repeated measures test was conducted to evaluate the impact of omeprazole on gastrin and serum cobalamin levels over a 60-day administration period. The sphericity condition was analyzed using the Mauchly test. In cases of non-normality, non-parametric tests were utilized, specifically the Friedman test with Wilcoxon post hoc contrasts. The significance level was adjusted to 0.05/3 = 0.017 (Bonferroni correction). The plots were created using Matplotlib, a data visualization library in Python developed by the Matplotlib community.

## 3. Results

### 3.1. Study Population

Finally, 11 spayed females, 2 neutered males, and 5 intact males were included. The age range of the dogs varied from 8 months to 8 years, with a mean age of 3.77 years. Eleven mixed-breed dogs were included: 2 American Staffordshire Terrier, 2 Greyhounds, 1 Hound, 1 Dalmatian, and 1 Weimaraner. The mean weight was 11.9 kg, the body score of all dogs assessed by the body score scale (1 to 9) was between 4 and 5, and the muscular condition was normal in all the selected samples.

### 3.2. Serum Gastrin Levels

**Figure 2:**
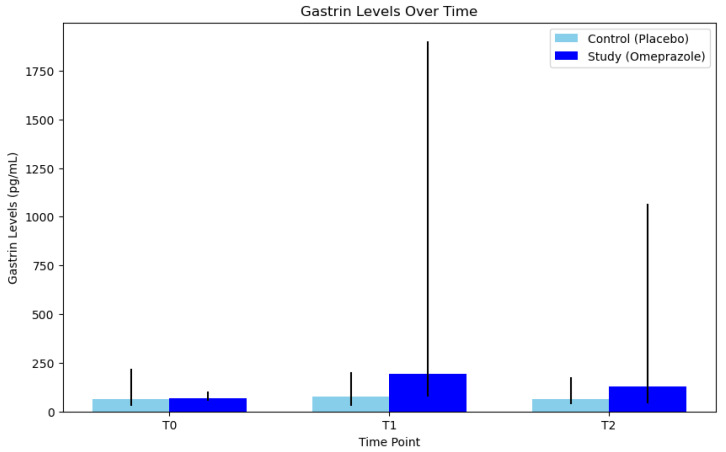
Comparison of serum gastrin levels between the control group (placebo) and the study group (omeprazole), at times T_0_ (day 0), T_1_ (day 30), and T_2_ (day 60).

The control group (placebo) showed outliers at T_0_, whereas the study group (omeprazole) exhibited outliers at T_1_ and T_2_. Due to the absence of normality in both groups, the non-parametric Friedman test was employed, setting the significance level at α = 0.017. In the control group, the median serum gastrin levels (pg/mL) were 62.45 [30.17–218.75] at T_0_, 76.06 [30.67–199.87] at T_1_, and 63.02 [35.81–176.06] at T_2_. Conversely, the study group demonstrated median serum gastrin levels of 67.59 [55.96–101.60] at T_0_, 191.77 [75.31–1901.77] at T_1_, and 128.16 [43.62–1066.46] at T_2_. Significant differences were observed within the study group (*p* = 0.008) (Figure 2).

In the study group, between T_0_ and T_1_, no statistically significant differences were observed (*p* = 0.138); nevertheless, substantial disparities were noted (*p* = 0.001) in the study group. Between T_1_ and T_2_ no statistically significant differences were detected in either the control group (*p* = 0.423) or the study group (*p* = 0.422). Between T_0_ and T_2_, in the control group, no statistically significant differences were observed (*p* = 0.313). Conversely, in the study group, significant differences were noted between T_0_ and T_2_ (*p* = 0.012).

### 3.3. Serum Cobalamin Levels

**Figure 3:**
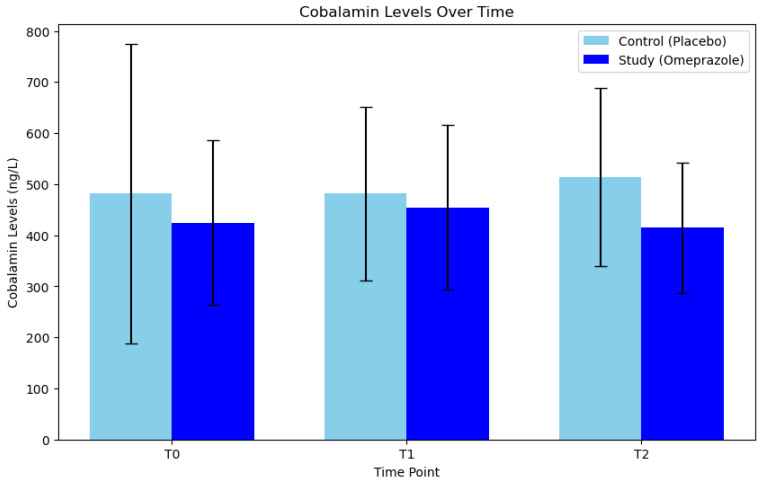
Comparison of serum cobalamin levels between the control group (placebo) and the study group (omeprazole), at times T_0_ (day 0), T_1_ (day 30), and T_2_ (day 60).

In the control group, one outlier was identified and subsequently removed at T_0_, while no outliers were detected in the study group. The mean cobalamin levels (ng/L) in the control group were 481.4 (±293.70) at T_0_, 481.4 (±170.21) at T_1_, and 513.2 (±174.50) at T_2_. In the absence of sphericity, the Greenhouse-Geisser correction was used, resulting in a *p*-value of 0.714, indicating no significant differences in pairwise comparisons based on the timing of placebo administration in dogs’ serum cobalamin levels. Conversely, the study group exhibited cobalamin levels of 424.62 (±161.57) at T_0_, 454.5 (±160.96) at T_1_, and 414.8 (±127.90) at T_2_. In this group, the *p*-value was 0.305, indicating that treatment with omeprazole did not induce differences in cobalamin levels across different administration times (Figure 3). 

### 3.4. Adverse Effects Related to Omeprazole Administration

In the omeprazole group, 2 out of 11 dogs exhibited gastrointestinal disorders. One dog presented signs of nausea and vomiting during the initial two weeks of the study, followed by a brief period of diarrhea in the 5th week (with a fecal score of 6/6). Despite these symptoms, this participant remained in the study as the weight loss was less than 10%. Conversely, another dog developed severe gastrointestinal signs during the first week of treatment, including profuse diarrhea with a fecal score of 6/6 and hematochezia, consequently leading to its withdrawal from the study.

The median fecal score evaluated once per week by the owners was in the control group 3.5 (±0.50), whereas in the study group, it was 3.5 (±1.28).

## 4. Discussion

Proton pump inhibitors (PPIs) are effective when appropriately used for treating gastrointestinal acid-related disorders in human and veterinary medicine. However, multiple studies have identified patterns of inappropriate prescription characterized by the overuse of PPIs [18,19,20]. This study confirms that dogs chronically administered omeprazole develop hypergastrinemia but do not exhibit abnormalities in serum cobalamin concentrations. The use of PPIs is not recommended except for specific disease processes outlined in the ACVIM consensus statement, such as reflux or erosive esophagitis, gastrointestinal bleeding, acute abdomen secondary to gastroduodenal ulceration and erosion, gastric or duodenal perforation, prophylaxis of mast cell tumor or gastrinoma, and toxicosis associated with non-steroidal anti-inflammatory drugs (NSAIDs) [1].

The secretion of gastric acid is a normal physiological mechanism that contributes to the proper digestion of proteins, the release of cobalamin, the absorption of inorganic iron [21] and calcium [22], and the regulation of the intestinal microbiome by suppressing excessive bacterial growth [23]. The stomach has natural protective mechanisms to prevent gastric erosion and ulceration as a result of gastric acid secretion, and therefore, acid suppression is generally not recommended in the absence of erosive or infiltrative disease [20,24,25,26]. Routine administration of PPIs appears to occur frequently in the hospital setting, even for the treatment of nausea, vomiting, or both, despite there being no pharmacological evidence of an antiemetic effect or control of nausea [27]. It is also important to emphasize the side effects detected with their use, primarily detected when used long-term [2,4,14,27]. In this study, long-term administration of omeprazole was associated with increased serum gastrin levels. This finding is consistent with previous research indicating that omeprazole treatment leads to an increase in gastric pH by inhibiting the H^+^/K^+^ ATPase pump, resulting in reduced gastric acid production [5]. Gastrin increases because of the lack of negative feedback due to the pharmacological suppression of gastric acid [28]. Hypergastrinemia can potentially induce hyperplasia of enterochromaffin cells, which secrete histamine, stimulating gastric acid release from parietal cells [7].

In human medicine, hypergastrinemia related to the use of PPIs occurs in 80–100% of the cases [29] and may be a pathogenic factor in the development of gastric carcinoma [30]. Animal model studies on gastric hypoacidity and hypergastrinemia have yielded evidence suggesting hypergastrinemia as a common causative factor across various gastric pathologies. In species where sufficient hypoacidity and hypergastrinemia have been induced, a subset of animals develops malignant lesions in the gastric oxyntic mucosa [31]. Long-term administration of the proton pump inhibitor omeprazole in rats has been linked to hyperplasia of the oxyntic mucosa and carcinoids. Similarly, short-term omeprazole administration (400 μmol/kg) to rats led to a 15-fold increase in plasma gastrin levels, along with oxyntic mucosa hyperplasia [32,33,34]. Gastrin receptors in the oxyntic mucosa are predominantly found in enterochromaffin-like cells, which play a functional role in mucosal growth regulation [31]. Further investigations are warranted to elucidate the potential relationship between the long-term administration of PPIs and mucosa hyperplasia or gastric cancer in dogs and cats.

A study involving 231 dogs with chronic enteropathy receiving antisecretory therapy demonstrated higher levels of serum gastrin in dogs treated with omeprazole. However, serum gastrin levels in these dogs did not exceed 3 times the upper reference limit (URL), excluding the diagnosis of gastrinoma [13]. Notably, the omeprazole group in the present study exhibited mean gastrin levels four times higher than the control group. This could be due to the variability between laboratories, or variability between the different times in which the samples were analyzed. Nonetheless, gastric histological samples were not evaluated in this study.

A study involving six cats showed that long-term PPI use resulted in increased serum gastrin levels in 83.33% of the sample after 30 days and in 100% of the sample after 60 days [7]. In this study, 100% of dogs exhibited increased serum gastrin levels after 30 days of treatment, with 87.7% showing increased levels after 60 days. This outcome is consistent with the more pronounced hypergastrinemia observed in the initial phase in other studies involving dogs treated with famotidine, where a notable increase in gastrin levels shortly after treatment initiation was detected, followed by a gradual decrease in serum gastrin levels from the second week of treatment onwards [1,20].

In this study, no statistical differences were found in cobalamin during the long-term administration of omeprazole compared to the control group. Nevertheless, in human medicine, decreases in cobalamin levels have been reported in patients undergoing long-term PPI therapy [35,36]. Dose-dependent decreases have also been noted, with differences observed between the administration of 20 mg or 40 mg capsules [37]. Conversely, more recent studies have not found an association between long-term PPI therapy and decreased cobalamin absorption [38,39].

There are no previous studies that relate the use of PPIs with the decrease in cobalamin levels in dogs. In cats, one study evaluated changes in cobalamin levels during long-term omeprazole therapy and found no significant differences in cobalamin serum levels [7]. As the relationship between PPI therapy and cobalamin levels remains controversial in human medicine, and the present study did not detect any changes, it is plausible that cobalamin serum levels may remain stable with long-term omeprazole therapy in dogs. Nevertheless, further studies involving larger populations and longer durations are warranted to validate these findings.

In this study, 18.18% of the sample in the omeprazole group exhibited gastrointestinal signs such as diarrhea, contrasting with the control group. There was a higher median fecal score in the study group compared to the control group. This suggests that long-term administration of omeprazole could induce changes in the gastrointestinal microbiome, as described in other studies [1]. Diarrhea is the most common adverse effect associated with PPI administration in veterinary medicine; however, the mechanism is not fully understood. Following a 15-day treatment with omeprazole, dogs exhibited an increase in the canine fecal microbiota dysbiosis index [14]. It is believed that the effect of PPIs on the gastrointestinal microbiome is partly due to their impact on gastric pH [40]. Additionally, genetic factors such as variability in CYP_450_ expression could result in differences in PPI metabolism among dogs, leading to diarrhea symptoms in some animals but not in others [10].

A veterinary medicine study conducted in healthy dogs receiving omeprazole twice daily for 15 days revealed significant alterations in the gastrointestinal microbiome. [14]. Although the microbiome was not a study variable in this research, it is possible that side effects such as diarrhea in 2 out of 11 dogs were due to intestinal dysbiosis. Small intestinal dysbiosis is another widely described adverse effect of chronic PPI administration in humans [41,42]. PPIs increase the survival of ingested bacteria in the upper gastrointestinal tract by reducing intestinal peristalsis, gastric emptying, altering the composition of epithelial mucus, increasing pH, and promoting bacterial translocation. Increased bacterial growth in the upper gastrointestinal tract may elevate the risk of bacterial pneumonia by aspiration [1,17].

The main limitations of the current study include the restricted sample size and the absence of a crossover design, which represents an advantage, especially in situations where interindividual variability can be a significant factor to consider. Changes in cobalamin levels observed in humans occurred with longer administrations than the duration of our study. Hence, a more extended administration period may be necessary to assess changes in intestinal absorption of this vitamin with long-term PPI use. Other complications observed in human medicine due to the use of this medication, such as abnormalities in serum iron, calcium, and magnesium levels, leading to alterations in patients’ ossification with long-term administration, have been reported. Due to economic constraints in the present study, these parameters could not be evaluated, but it would be interesting to assess their implications in future research. Additionally, evaluating the microbiome and parameters of intestinal inflammation, such as calprotectin assessment, would have been beneficial to discern the degree of involvement of PPI-associated dysbiosis in our study.

## 5. Conclusions

The long-term administration of omeprazole in dogs significantly increases serum gastrin levels, but no significant changes have been found in serum cobalamin levels. The identification of increased gastrin levels within this population emphasizes the critical need for a comprehensive clinical evaluation of potential related gastrointestinal disorders, with special attention given to the use of omeprazole as a chronic preventive treatment in dogs. Side effects such as diarrhea or vomiting have been present in 18% of the sample. These complications could be associated with the development of gastrointestinal dysbiosis, as has been widely described in previous studies in both human and veterinary medicine.

## Data Availability

Data are contained within the article.
